# Difference in adult food group intake by sex and age groups comparing Brazil and United States nationwide surveys

**DOI:** 10.1186/1475-2891-13-74

**Published:** 2014-07-21

**Authors:** Ilana Nogueira Bezerra, Joseph Goldman, Donna G Rhodes, Mary Katherine Hoy, Amanda de Moura Souza, Deirdra N Chester, Carrie L Martin, Rhonda S Sebastian, Jaspreet K Ahuja, Rosely Sichieri, Alanna J Moshfegh

**Affiliations:** 1 Centro de Ciências da Saúde, Curso de Nutrição, Universidade de Fortaleza (UNIFOR), Av. Washington Soares 1321, sala C04 Edson Queiroz, Fortaleza, Brazil; 2 Food Surveys Research Group, Beltsville Human Nutrition Research Center, Agricultural Research Service, USDA, 10300 Baltimore Ave. Building 005, BARC West, 20705 Baltimore, Beltsville, MD, USA; 3 Department of Epidemiology, Institute of Social Medicine, State University of Rio de Janeiro, Rua São Francisco Xavier, 524, 7° andar, Bloco E, Cep 20550–012, Rio de Janeiro, RJ, Brazil; 4 United States Department of Agriculture, National Institute of Food and Agriculture, 1400 Independence Avenue SW, Stop 2201, Washington, DC, 20250–2201, USA

**Keywords:** Diet, Food groups, Dietary surveys, Brazilian and American diet, Food habits

## Abstract

**Background:**

International comparisons of dietary intake are an important source of information to better understand food habits and their relationship to nutrition related diseases. The objective of this study is to compare food intake of Brazilian adults with American adults identifying possible dietary factors associated with the increase in obesity in Brazil.

**Methods:**

This research used cross-national analyses between the United States and Brazil, including 5,420 adults in the 2007–2008 What We Eat In America, National Health and Nutrition Examination Survey and 26,390 adults in the 2008–2009 Brazilian Household Budget Survey, Individual Food Intake. Dietary data were collected through 24 h recalls in the U.S. and through food records in Brazil. Foods and beverages were combined into 25 food categories. Food intake means and percentage of energy contribution by food categories to the population’s total energy intake were compared between the countries.

**Results:**

Higher frequencies of intake were reported in the United States compared to Brazil for the majority of food categories except for meat, rice and rice dishes; beans and legumes; spreads; and coffee and tea. In either country, young adults (20-39 yrs) had greater reports of meat, poultry and fish mixed dishes; pizza and pasta; and soft drinks compared to older adults (60 + yrs). Meat, poultry and fish mixed dishes (13%), breads (11%), sweets and confections (8%), pizza and pasta (7%), and dairy products (6%) were the top five food category sources of energy intake among American adults. The top five food categories in Brazil were rice and rice dishes (13%), meat (11%), beans and legumes (10%), breads (10%), and coffee and tea (6%). Thus, traditional plant-based foods such as rice and beans were important contributors in the Brazilian diet.

**Conclusion:**

Although young adults had higher reports of high-calorie and nutrient-poor foods than older adults in both countries, Brazilian young adults did not consume a diet similar to Americans, indicating that it is still possible to reverse the current trends of incorporating Western dietary habits in Brazil.

## Background

International comparisons of dietary intake are an important source of information to better understand food habits and their relationship to nutrition related diseases, and contribute to the implementation of public health policies that address food intake, eating behavior and food environmental factors. However, comparisons of individual dietary intake across nations are infrequently reported in the literature [1]. Data from Brazil on household food availability indicate changes in the last three decades toward a Western dietary pattern. The availability of processed foods including soft drinks and cookies has increased more than 400 percent as compared to a small decrease in the availability of rice and beans [2,3]. These changes have been associated with the emergence of overweight and obesity in developing countries [4,5].

However, there are no data comparing the consumption of foods in Brazil to countries with an established Western dietary pattern, such as the United States. Although the proportion of Brazilians who are overweight has increased in the last decade, it is still less than the proportion of overweight Americans. The prevalence of obesity among adults in the United States was 34% in 2007-2008, compared to 15% in Brazil in 2008-2009 [6,7].

The increase in overweight between 2002 and 2008 was greater among Brazilian adults 20–44 years old compared to adults over 45 years old [8,9]. Previous research suggests that younger adults are more prone to important shifts in health behavior patterns, such as declines in overall diet quality and physical activity habits, that may have important impacts on long-term health [10,11].

The differences between Brazil and the United States were not limited to obesity; the prevalence of nutrition-related conditions, such as hypertension and diabetes, was lower among Brazilian adults compared to American adults. In the United States, the prevalence of hypertension in adults was 29% in 2009-2010 [12]. In Brazil, the prevalence was 24% in 2012 [13]. For diabetes, the prevalence in the United States was 11% of adults in 2010 compared to 7% in Brazil in 2012 [13,14].

The objective of this study is to compare food intake of Brazilian adults with American adults in order to identify dietary factors that could explain the differences in the prevalence of obesity and other nutrition-related conditions between the two countries.

## Methods

### Data source

Data for this study were provided by participants from the What We Eat in America (WWEIA), National Health and Nutrition Examination Survey (NHANES) 2007–2008 and from the 2008–2009 Brazilian Household Budget Survey, Individual Food Intake (HBS-IFI).

The NHANES is a continuous nationally representative survey of the civilian, non-institutionalized United States population conducted by the National Center for Health Statistics (NCHS), Center for Disease Control (CDC), Department of Health and Human Services (DHHS). WWEIA is the dietary intake component of NHANES, conducted jointly by the Agricultural Research Service, U.S. Department of Agriculture (USDA) and the NCHS, DHHS. The Brazilian Household Budget Survey (HBS) is conducted every five years in a nationally representative sample of Brazilian families by the Brazilian Office of Geography and Statistics (Instituto Brasileiro de Geografia e Estatística - IBGE). In 2008–2009, an interview module of individual food intake was added to the survey and carried out along with the household survey.

The Brazilian survey was conducted under Federal Law (n. 5534, Nov 14, 1968) that guarantees confidentiality. Data collected in NHANES are protected according to Federal Law (the Public Health Service Act (42 USC 242 k), the Privacy Act of 1974 (5 USC 552A), and the Confidential Information Protection and Statistical Efficiency Act (PL 107–347)). Because the current study was a secondary analysis, no institutional review board approval was necessary.

### Sample

The NHANES sample utilizes a complex, multistage, probability sampling design. In the first stage, primary sampling units (PSU) were selected with probability proportional to its size. In the second stage, segments in each PSU were selected with probability proportional to a measure of size. Households within each segment were listed and randomly selected, and in the last stage individuals were drawn at random within designated age-sex-race/ethnicity screening subdomains from a list of all persons residing in the selected households. For the 2007–2008 NHANES, 12,943 persons were selected and 9,255 completed a one day dietary intake interview at the Mobile Examination Center.

The Brazilian HBS utilizes a two-stage sampling technique to select the participating households. In the first stage, PSUs were selected by systematic sampling with proportional probability to the number of households. In the second stage, households were selected by simple random sampling without replacement. The PSUs were stratified to be representative of all Brazilian regions, capitals of the 26 states, metropolitan areas, urban and rural areas, and several socioeconomic levels. The final sample of the HBS included 4,696 PSUs with 55,970 households. The dietary survey (HBS-IFI) was conducted in 25% of this sample, 13,573 households. Family members aged 10 years or older were included with a final sample of 34,003 individuals [15].

For this analysis, only dietary data from the first day of collection was included since a single day’s intake is sufficient to estimate population mean intake [16]. Analysis was limited to adults aged 20 years old and over who provided dietary data; thus, the final sample was 5,420 in the WWEIA, NHANES and 26,390 in the HBS-IFI. Data are reported for the following age groups: young adults (20–39 years old), middle-aged adults (40–59 years old) and older adults ( ≥ 60 years old). Sample size and characteristics of the participants of each survey are described in Table 1.

**Table 1:** **Sample population characteristics** - **means or percentages** (**standard error**) **in the United States**^**a **^**and Brazil**^**b**^

**Characteristic**	**UNITED STATES**			**BRAZIL**		
	**20-39y**	**40-59y**	**60 + y**	**20-39y**	**40-59y**	**60 + y**
	**(n = 1,751)**	**(n = 1,722)**	**(n = 1,947)**	**(n = 13,175)**	**(n = 8,893)**	**(n = 4,322)**
Women (%)	51.5 (1.4)	52.7 (1.3)	55.5 (1.1)	51.2 (0.5)	52.8 (0.6)	55.6 (0.9)
Age (y)	29.4 (0.3)	49.2 (0.2)	70.0 (0.3)	29.1 (0.1)	48.5 (0.1)^*^	69.7 (0.2)
Body mass index (kg/m^2^)^c^	28.0 (0.3)	29.2 (0.2)	28.8 (0.2)	24.8 (0.1)^*^	26.4 (0.1)^*^	26.3 (0.1)^*^
Daily energy intake (kJ/day)	9,471.3 (156.9)	9,192.2 (172.4)	7,308.2 (153.1)	8,372.6 (58.6)^*^	7,702.7 (69.0)^*^	6,860.5 (73.2)^*^

^a^Includes first day of intake, What We Eat in America, National Health and Nutrition Examination Survey 2007–2008.

^b^Includes first day of intake, Household Budget Survey, Individual Food Intake 2008–2009.

*Statistically different at p <0.05.

### Dietary data collection

In WWEIA, NHANES, 24-hour recalls are used to collect intakes of all foods and beverages, including water. The first dietary recall interview is collected in-person and the second is by telephone. Both recalls are conducted by trained interviewer using the U.S. Department of Agriculture Automated Multiple-Pass Method (AMPM) that uses 5 steps with multiple memory cues to accurately recall all possible foods and beverages consumed. The quick list is the first step when participants recall all foods and beverages, including water, consumed the day before the interview. In the second step, respondents are asked about commonly forgotten foods. Time and meal occasion are collected for each food in step three, a detailed description of each food, amount eaten, source and whether it was eaten at home are collected in step four. The last step provides a final opportunity to recall foods [17].

In HBS-IFI, food records were used to collect dietary intake for 2 non-consecutive days. The records comprised all food and beverages consumed, excluding water, amount consumed, place of the meal (at home or out of home), time of intake and preparation of food for specific items. Respondents were encouraged to record their intake with as much detail as possible. All participants received a booklet with instructions on how to fill in the records and with pictures and household measures for portion-size estimation. Interviewers reviewed all food records, probing for food details and usually forgotten items, and recorded them on a computer database. When a record showed more than 3 hours during the daytime without any food intake or if the respondents ate less than 5 items in a single day, the interviewer verified that nothing else was consumed.

### Food categories

Dietary intakes in WWEIA, NHANES were coded into more than 4000 different codes and dietary intakes in HBS-IFI were coded into 2000 different codes. For this analysis, food codes representing similar food and beverage items were combined into 25 mutually exclusive food categories. The categorization of the foods was guided by how foods are customarily consumed. A description of each food category is detailed in Table 2.

**Table 2:** Description of food categories

**Food categories**	**Types of foods in the category**
Milk	Whole, reduced fat, fat-free milk, flavored milk, milk substitutes
Dairy products	Cheeses, cottage, ricotta, yogurt, ice cream, cream
Meat	Beef, pork, lamb, game
Poultry	Poultry, chicken patties, nuggets
Fish and seafood	Finfish and shellfish
Eggs and egg dishes	Scrambled, fried, omelet, quiches, soufflés
Protein mixed dishes	Meat, poultry and fish items that contain grain and/or vegetables
Deli and cured meats	Ham, luncheon meats, frankfurters, bacon, sausage
Beans and legumes	Beans and lentils, bean dishes, soy, textured vegetable protein
Rice and rice dishes	Rice and rice dishes that contain sauces, meat and/or vegetables
Breads	Breads, Rolls, tortillas, biscuits, muffins, doughnuts, waffles, French toast
Savory snacks	Chips, popcorn, pretzels, crackers
Pizza and pasta	Pizza, calzones, pizza rolls, spaghetti, ravioli, macaroni, lasagna
Cereals and grains	Ready-to-eat and cooked cereals, grits, oatmeal
Sweets and confections	Chocolate candies, caramels, chewing gum, cookies, cakes, pies
Fruits	All fruits including berries, citrus, melons, dried fruit
Vegetables	All vegetables including dark green, red/orange, starchy, potatoes
Coffee and tea	Brewed and instant coffee, coffee drinks, brewed and bottled tea
Juices	Fruit and vegetable juices
Soft drinks	Regular and artificial sweetened carbonated beverages
Alcoholic beverages	Beer, wine, and other alcoholic beverages
Spreads	Butter, margarine, cream cheese, dips
Oils and nuts	Salad dressings, mayonnaise, oils, peanuts, nut butters, coconut
Sauces and condiments	Catsup, salsa, barbecue sauce, gravies, pickles/pickled vegetables
All other foods	Basic ingredients

To make the data from each survey more comparable, water intake and the consumption of sugar added to beverages were modified. Because water intake was not collected in HBS-IFI, water reports in WWEIA, NHANES were excluded from this analysis. In HBS-IFI, the consumption of sugar and sugar substitutes added to beverages was estimated by asking the respondents the type of sweetener usually added to beverages: sugar, non-caloric artificial sweeteners, both, or none. If the participant reported sugar or both sugar and non-caloric sweetener, a proportional amount of sugar was added to commonly sweetened-beverages, such as juices, tea, and coffee. A 10% sugar-dilution was applied to intakes of respondents who usually added sugar and a 5% sugar-dilution was applied to intakes of respondents who usually added both sugar and non-caloric sweetener. In WWEIA, NHANES, any addition to a food or beverage and the amount of the addition was asked and recorded. Therefore, any addition of sugar and non-caloric sweeteners were included in the intakes of juices, tea, and coffee.

### Data analyses

Results are analyzed separately by age and sex at the country level, using Statistical Analysis Software (version 9.1, 2003, SAS Institute Inc, Cary, NC). The frequency of foods consumed by category was calculated from the number of individuals having reported eating at least one of the food items within the category divided by the total. In order to evaluate the proportion of energy provided by each food category, percentages were estimated as a ratio of total energy from each food category for all individuals to total daily energy intakes for all individuals. T-statistics were used to compare the estimates between the countries.

Sample weights were applied to obtain nationally representative estimates, and the stratification and clustering of the design of each survey were incorporated into the analyses. The *domain* statement was used for subpopulation analyses and the *smsub* SAS macro was used to incorporate the domain statement into ratio analyses.

## Results

### Population characteristics

Table 1 describes the survey population characteristics of each survey. The proportion of women and mean age were similar between the countries. Body mass index (BMI) and mean daily energy intake of adults were higher in the United States than in Brazil. American adults between 20 and 59 years of age consumed an average of 836.8 kJ (200 kcal) more than Brazilian adults and those aged 60 years and over consumed an average of 418.4 kJ (100 kcal) more.

### Frequency of reporting foods

Dietary intakes in the United States showed higher frequencies of intake for a majority of the food categories, with frequencies twice those in Brazil for milk, dairy products, deli and cured meat, savory snacks, cereal and grains, sweets and confections, and soft drinks. For alcoholic beverages, the frequency was four times higher in the United States compared to Brazil (Table 3). However, Brazil had higher frequencies for meat, beans and legumes, rice and rice dishes, spreads, coffee and tea, and juices.

**Table 3:** **Frequency of reporting food categories**, **percent of total energy intake provided by food categories and p**-**values among adults** (**20** + **y**) **in the United States**^**a **^**and Brazil**^**b**^

**Food categories**	**UNITED STATES**		**BRAZIL**		**p-values**	
	**% reporting**	**% of energy**	**% reporting**	**% of energy**	**% reporting**	**% of energy**
Milk	46.9	3.7	23.1	2.5	<0.0001	<0.0001
Dairy products	62.4	5.9	17.8	2.0	<0.0001	<0.0001
Meat	26.1	3.6	54.5	11.2	<0.0001	<0.0001
Poultry	25.9	3.6	27.3	4.7	0.2756	<0.0001
Fish and seafood	9.8	1.1	8.2	2.0	0.0356	<0.0001
Eggs and egg dishes	20.5	2.0	15.7	1.3	0.0006	<0.0001
Protein mixed dishes	53.8	12.6	33.0	4.9	<0.0001	<0.0001
Deli and cured meats	36.0	3.3	16.3	1.4	<0.0001	<0.0001
Beans and legumes	11.4	1.1	77.5	10.4	<0.0001	<0.0001
Rice and rice dishes	15.4	1.9	89.2	12.5	<0.0001	<0.0001
Breads	76.0	10.5	70.1	9.7	<0.0001	0.0111
Savory snacks	44.1	4.4	18.6	1.8	<0.0001	<0.0001
Pizza and pasta	25.1	6.9	22.8	4.2	0.0931	<0.0001
Cereals and grains	32.8	3.2	12.4	1.4	<0.0001	<0.0001
Sweets and confections	60.8	7.5	32.4	5.5	<0.0001	<0.0001
Fruits	44.1	2.6	36.1	3.1	0.0005	0.0092
Vegetables	69.6	5.4	56.7	3.0	<0.0001	<0.0001
Coffee and tea	66.2	1.9	85.6	6.2	<0.0001	<0.0001
Juices	32.8	3.0	38.7	5.2	0.0026	<0.0001
Soft drinks	58.3	5.4	23.3	1.8	<0.0001	<0.0001
Alcoholic beverages	23.4	4.4	5.0	1.1	<0.0001	<0.0001
Spreads	29.1	1.1	40.0	2.4	<0.0001	<0.0001
Oils and nuts	43.4	3.8	2.5	0.1	<0.0001	<0.0001
Sauces and condiments	46.7	1.0	1.8	0.1	<0.0001	<0.0001
All other foods	2.4	0.2	11.8	1.4	<0.0001	<0.0001

^a^Includes first day of intake, What We Eat in America, National Health and Nutrition Examination Survey 2007–2008.

^b^Includes first day of intake, Household Budget Survey, Individual Food Intake 2008–2009.

Although Brazil had a higher frequency for spreads, it was not statistically different between the countries for older adults. No differences were found between the countries for poultry, and pizza and pasta consumption with the exception of poultry in older adults. The frequency of reporting fruits was similar between the countries with the exception of woman 60 years and over which was higher in the United States (Tables 4 and 5).

**Table 4:** **Frequency of reporting food categories and percent of total energy intake provided by food categories among men in the United States**^**a **^**and Brazil**^**b**^, **according to age**

**Food categories**	**UNITED STATES**						**BRAZIL**					
	**20**-**39y (n = 860)**		**40**-**59y (n = 843)**		**60 + y (n = 959)**		**20**-**39y (n = 5,930)**		**40**-**59y (n = 4,044)**		**60 + y (n = 1,993)**	
	**% reporting**	**% of energy**	**% reporting**	**% of energy**	**% reporting**	**% of energy**	**% reporting**	**% of energy**	**% reporting**	**% of energy**	**% reporting**	**% of energy**
Milk	40.1	3.2	46.0	3.4	55.6	4.2	23.1^*^	2.4	18.9^*^	1.8^*^	21.4^*^	2.3^*^
Dairy products	56.5	4.6	63.8	5.6	62.0	6.2	16.7^*^	1.7^*^	15.6^*^	1.7^*^	17.3^*^	2.0^*^
Meat	27.0	4.1	32.2	4.3	26.9	3.7	59.1^*^	11.6^*^	59.5^*^	12.3^*^	53.2^*^	11.5^*^
Poultry	28.3	4.0	27.8	3.8	19.6	2.6	28.3	4.6	27.4	4.8	26.2^*^	4.4^*^
Fish and seafood	10.1	0.9	10.2	1.4	10.8	1.4	8.3	2.0^*^	8.4	2.1	8.4	2.4^*^
Eggs and egg dishes	19.7	1.8	23.9	2.3	26.8	2.5	17.8	1.4	17.8^*^	1.4^*^	15.8^*^	1.4^*^
Protein mixed dishes	56.9	14.0	55.1	12.6	54.8	12.4	35.3^*^	5.1^*^	32.2^*^	4.5^*^	30.2^*^	4.5^*^
Deli and cured meats	34.7	3.4	40.7	4.1	45.6	4.0	19.8^*^	1.6^*^	17.2^*^	1.5^*^	14.1^*^	1.5^*^
Beans and legumes	12.7	1.1	11.8	1.0	11.4	1.4	81.2^*^	11.0^*^	83.1^*^	12.1^*^	81.2^*^	11.6^*^
Rice and rice dishes	18.8	2.3	16.4	2.0	12.9	1.6	91.0^*^	13.3^*^	91.1^*^	13.6^*^	87.4^*^	13.0^*^
Breads	72.8	9.6	81.2	10.8	82.9	11.2	68.7	9.2	69.0^*^	9.3^*^	68.4^*^	9.8^*^
Savory snacks	42.4	4.2	43.5	4.0	40.8	3.8	15.7^*^	1.8^*^	13.7^*^	1.1^*^	17.0^*^	1.3^*^
Pizza and pasta	31.3	9.3	23.7	6.5	16.1	4.2	27.2	4.7^*^	21.8	4.1^*^	17.8	2.9^*^
Cereals and grains	26.0	2.5	26.2	2.5	42.5	4.2	11.5^*^	1.3^*^	10.7^*^	1.2^*^	14.6^*^	1.9^*^
Sweets and confections	49.5	5.2	57.2	7.7	66.5	8.6	28.8^*^	5.0	27.3^*^	4.2^*^	30.1^*^	4.5
Fruits	35.3	1.9	39.1	1.9	55.5	3.7	26.3^*^	2.1	32.7	2.8^*^	39.7^*^	4.0^*^
Vegetables	61.2	4.4	69.3	5.5	74.0	6.4	51.9^*^	2.8^*^	59.5^*^	3.1^*^	58.4^*^	3.4^*^
Coffee and tea	48.5	1.6	70.2	1.9	84.3	1.5	78.6^*^	4.9^*^	89.7^*^	6.2^*^	92.2^*^	7.2^*^
Juices	37.4	3.4	29.7	2.5	35.3	2.5	41.9	5.5^*^	34.6	4.4^*^	30.8	3.8^*^
Soft drinks	69.4	7.6	62.7	5.2	48.1	2.8	31.1^*^	2.4^*^	22.0^*^	1.6^*^	14.1^*^	0.9^*^
Alcoholic beverages	34.5	6.0	28.8	5.5	26.5	4.3	7.2^*^	1.5^*^	9.9^*^	2.5^*^	6.9^*^	1.6^*^
Spreads	19.6	0.7	28.8	1.1	35.8	1.5	38.7^*^	2.2^*^	36.7^*^	2.3^*^	36.2	2.3^*^
Oils and nuts	37.4	3.1	43.7	3.5	44.2	4.4	2.5^*^	0.1^*^	2.3^*^	0.1^*^	2.5^*^	0.1^*^
Sauces and condiments	50.9	1.0	51.3	1.0	46.9	0.8	1.9^*^	#^*^	2.1^*^	0.1^*^	1.3^*^	#^*^
All other foods	3.9	0.4	1.6	0.1	2.8	0.2	14.1^*^	1.7^*^	12.5^*^	1.5^*^	14.1^*^	2.0^*^

^a^Includes first day of intake, What We Eat in America, National Health and Nutrition Examination Survey 2007–2008.

^b^Includes first day of intake, Household Budget Survey, Individual Food Intake 2008–2009.

#indicates a non-zero estimates too small to display.

*Statistically different at p <0.05.

**Table 5:** **Frequency of reporting food categories and percent of total energy intake provided by food categories among women in the United States**^**a **^**and Brazil**^**b**^, **according to age**

**Food categories**	**UNITED STATES**						**BRAZIL**					
	**20-39y (n = 891)**		**40-59y (n = 879)**		**60 + y (n = 988)**		**20-39y (n = 7,245**)		**40-59y (n = 4,849)**		**60 + y (n = 2,329)**	
	**% reporting**	**% of energy**	**% reporting**	**% of energy**	**% reporting**	**% of energy**	**% reporting**	**% of energy**	**% reporting**	**% of energy**	**% reporting**	**% of energy**
Milk	41.8	3.7	46.8	3.9	58.0	4.5	25.0^*^	2.9^*^	22.4^*^	2.4^*^	28.6^*^	3.4^*^
Dairy products	59.9	6.5	65.2	6.4	67.9	7.8	17.5^*^	2.1^*^	19.9^*^	2.3^*^	21.0^*^	2.5^*^
Meat	20.0	2.5	26.6	3.4	23.9	2.8	51.6^*^	10.6^*^	52.3^*^	10.7^*^	47.1^*^	9.9^*^
Poultry	27.5	3.8	27.1	3.3	20.7	2.7	29.1	5.0^*^	25.0	4.4^*^	25.0	4.5^*^
Fish and seafood	9.4	0.9	9.2	1.0	9.6	1.3	7.9	1.9^*^	8.4	2.1^*^	8.0	2.4^*^
Eggs and egg dishes	16.9	1.7	17.8	1.6	21.0	2.1	15.0	1.2	14.4	1.2^*^	11.2^*^	1.1^*^
Protein mixed dishes	53.9	12.9	52.2	11.7	49.5	10.9	32.7^*^	4.8^*^	32.9^*^	5.1^*^	32.3^*^	4.7^*^
Deli and cured meats	32.6	2.9	30.7	2.2	37.0	2.8	16.8^*^	1.3^*^	14.2^*^	1.2^*^	10.4^*^	1.0^*^
Beans and legumes	10.8	0.9	11.0	1.1	11.0	1.2	72.3^*^	8.8^*^	74.6^*^	9.6^*^	74.4^*^	9.5^*^
Rice and rice dishes	17.5	2.1	14.7	1.7	9.2	1.1	87.9^*^	11.5^*^	87.7^*^	11.6^*^	89.3^*^	11.9^*^
Breads	68.5	10.5	74.8	10.5	80.7	11.9	70.5	10.0	73.3	10.5	70.0^*^	10.4^*^
Savory snacks	43.2	4.5	49.4	5.3	43.2	4.2	21.9^*^	2.1^*^	21.9^*^	2.0^*^	20.6^*^	2.6^*^
Pizza and pasta	31.0	7.4	24.1	6.6	18.3	4.5	25.1	4.7^*^	20.8	4.2^*^	15.7	2.9^*^
Cereals and grains	28.0	3.1	34.2	3.7	48.2	5.2	12.8^*^	1.5^*^	11.7^*^	1.4^*^	16.4^*^	2.1^*^
Sweets and confections	59.5	7.0	65.7	8.8	71.3	10.1	38.5^*^	6.9	31.9^*^	6.2^*^	36.8^*^	6.0^*^
Fruits	36.1	2.3	46.6	3.0	62.3	4.8	36.4	3.0^*^	41.8	3.7^*^	52.1^*^	5.2
Vegetables	67.8	5.4	72.9	5.5	75.8	6.5	54.0^*^	2.9^*^	60.3^*^	3.3^*^	62.2^*^	3.4^*^
Coffee and tea	49.9	2.4	74.2	2.4	82.3	1.5	82.2^*^	6.1^*^	91.0^*^	7.7^*^	88.8^*^	7.5^*^
Juices	33.2	3.4	27.4	2.8	37.2	2.8	45.3^*^	6.4^*^	36.4^*^	5.0^*^	31.6	4.1^*^
Soft drinks	62.4	6.9	58.2	4.4	39.2	2.5	26.1^*^	2.0^*^	19.7^*^	1.4^*^	13.5^*^	0.8^*^
Alcoholic beverages	18.8	3.3	17.8	3.5	13.3	1.9	2.2^*^	0.4^*^	2.3^*^	0.4^*^	1.4^*^	0.2^*^
Spreads	27.7	1.0	29.2	1.1	39.3	1.7	42.3^*^	2.6^*^	42.7^*^	2.6^*^	40.2	2.6^*^
Oils and nuts	43.6	4.0	46.2	4.3	46.2	4.3	2.5^*^	0.1^*^	2.8^*^	0.2^*^	2.3^*^	0.1^*^
Sauces and condiments	48.7	1.1	43.8	1.3	36.1	0.8	1.7^*^	0.1^*^	1.8^*^	0.1^*^	1.6^*^	0.1^*^
All other foods	2.2	0.1	1.6	0.1	2.5	0.1	11.1^*^	1.2^*^	9.5^*^	1.2^*^	9.4^*^	1.2^*^

^a^Includes first day of intake, What We Eat in America, National Health and Nutrition Examination Survey 2007–2008.

^b^Includes first day of intake, Household Budget Survey, Individual Food Intake 2008–2009.

*Statistically different at p < 0.05.

In the United States, the frequency of intakes of deli and cured meats was higher among older adults; in Brazil young adults had the highest frequency. The same was observed for the consumption of spreads, sweets and confections, and juices among women. In either country, young adults presented higher frequencies of reporting meat, poultry and fish mixed dishes, pizza and pasta, and soft drinks compared to older adults, who in contrast reported more fruits, vegetables, cereals and grains, and coffee and tea.

Among Brazilian men, the frequency of reporting milk was higher in young adults, while among American men the frequency was higher in older adults. Compared to men, women had higher frequencies of reporting savory snacks, milk, dairy products, fruits, vegetables, cereals and grains, and sweets and confections in both countries and in all age groups.

### Food sources of energy

The difference in energy intake between the countries was reflected in the contribution of energy provided by specific food categories (Table 3). Meat, poultry and fish mixed dishes; breads; sweets and confections; pizza and pasta; and dairy products were the top five food category sources of energy intake among American adults, contributing to 44% of total energy intake. The Brazilian diet included important contribution from traditional plant-based foods: rice (12.5%) and beans (10.4%). Plain meat, poultry, and fish and seafood were greater contributors to total energy intake than mixed dishes containing meat, poultry and fish in Brazil (18 versus 5%) compared to the United States (8 versus 13%). The consumption of breads was an important contributor to total energy intake in the United States and Brazil (10.5 and 9.7%, respectively).

Differences were seen in the consumption of beverages. In the United States, soft drinks contributed more to the total energy intake than in Brazil. The opposite was seen with coffee and tea intake, which provided three times more energy in Brazil than in the United States. The consumption of juices contributed proportionately more energy for Brazilian than for Americans.

Differences were also found in the contribution of food categories to total energy intake by gender and age (Tables 4 and 5). Although the frequency of reporting fruits was higher in the United States compared to Brazil, the contribution to total energy intake was higher in Brazil, except for older women and young men.

The contribution of sweets and confections to energy was higher among older adults in the United States and among young adults in Brazil. In both countries, energy from meat, poultry and fish mixed dishes, and savory snacks was higher among young men; energy from milk was higher among older women. The energy from sweets and confections, cereals and grains, fruits, vegetables, milk, dairy products, savory snacks, coffee and tea, spreads, and juices was higher among women than men.

In both countries, the energy from pizza and pasta, juices and soft drinks was higher among young adults, while the energy from breads, dairy products, fruits, vegetables, and cereals and grains was higher among older adults.

## Discussion

Important differences in dietary intakes of adults were observed comparing national dietary data from the United States and Brazil. Although young adults reported higher frequencies of high-calorie and nutrient-poor foods than older adults in both countries, young Brazilian adults still include traditional Brazilian foods in their diets.

The frequency of consumption of pasta and pizza and soft drinks decreased with age in both countries, while cereals and grains, fruit and vegetables increased with age. Young adults experience important changes in their life, such as initiating graduate school, leaving parents’ house, and starting to work. These changes are likely to impact health-related behaviors, including decreased overall diet quality [10,11]. In Brazil, the majority of young adults reported consuming rice and beans, suggesting that they keep the traditional Brazilian dietary pattern, but have also incorporated high-calorie and nutrient-poor foods into their diet.

The higher frequency of individuals reporting across food categories among American adults may be an indication of a greater variety of foods in their diet compared to those in Brazil. The frequency of reporting meat, poultry and fish mixed dishes in the United States was higher than in Brazil, while the frequency of reporting plain meat and poultry was higher in Brazil. Similar results were found by Kim and colleagues comparing American and Chinese diets. In the United States, the overall food variety, and the variety in protein sources were much higher than in China [18].

Chun and colleagues (2010) showed significant increases in the consumption of mixed dishes in the diets of American adults in the last three decades [19]. In Brazil, a limited variety diet that has relied on the consumption of rice and beans has been already identified as a traditional dietary pattern among Brazilian adults and a protection factor for obesity [20-23].

A varied diet may improve intake of some nutrients, but it can also be associated with a higher energy intake, overweight and obesity, if high-energy dense foods are incorporated [3,24-27]. The traditional dietary pattern in Brazil, characterized by high consumption of staple and basic foods, has been positively associated with a reduced BMI, and lower risk of cardiovascular diseases [23,28]. On the other hand, Western diets, characterized by a high consumption of high-calorie and nutrient-poor foods , have been associated with increasing prevalence of overweight and other adverse impacts on health [29,30].

The consumption of deli and cured meats, savory snacks, pizza and pasta, dairy products, and soft drinks was greater in the United States than in Brazil. The predominance of these foods in the American diet has already been observed by Block in previous surveys [31]. In Brazil, the consumption of a traditional dietary pattern was inversely associated with higher levels of education and income [22,32]. The relationship between income and diet is well known and as countries increase their economy, diets become more diverse and more people have access to a wider variety of food [33]. A recent paper using data from the same survey found that the prevalence of nutrient inadequacy declined with increasing income and education levels [34]. To compare Brazilian and American diets in order to identify whether similarities in the diet of these countries would explain the prevalence of obesity, additional analyses based on the consumption of food over time would be necessary.

Unfortunately, there is no time trend based on individual food intake in a representative Brazilian population. Data based on food expenditure suggest that the nutritional transition process in Brazil is an ongoing phenomenon characterized by important changes in the diet, such as the increasing availability of ultra-processed foods along with the decrease of household availability of traditional staple foods [3,35]. Pereira and colleagues (2014) evaluated the consumption of solid fats and added sugar (SoFAS), which include mostly processed foods and mixed dishes cooked with high contents of fats and/or sugar, and found that SoFAS contributed 52% of total energy intake in Brazil. They also observed the highest intakes of SoFAS among individuals in the highest quartile of per capita family income [36]. This scenario suggests that if the country continues this trend, it could potentially reach the same high levels of overweight and other nutrition related diseases as the United States. It is important to note that the increasing prevalence of overweight and obesity among developing countries has been attributed not only to changes in the diet, but also to changes in other lifestyle behaviors, such as physical inactivity [5].

The consumption of soft drinks contributed 5% to total energy intake in the United States and only 2% in Brazil. Soft drinks are one of the major sources of sugar in the American diet and also contribute to the intake of sugar in Brazil with availability dramatically increasing in the last decade [2,19,23]. Brazil is the highest sugar producer in the world and the second highest consumer of sugar in the world, after the United States [37]. The high energy contribution from coffee and tea in Brazil reveals that these beverages are also important drivers of sugar intake. Eighty-three percent of the respondents reported sugar as the sweetener usually added to beverages. The use of a standardized question to estimate sugar consumption could be a limitation in the amount of sugar consumed, since people could have used more or less sugar than the dilution considered in the recipes. Both the frequency of reporting the consumption of juices and the contribution to total energy intake differed between Brazil and the United States (except in men), indicating that the sugar added to juices is another important driver of energy intake in Brazil. It is important to note that most juices in the United States are consumed without adding table sugar.

A limitation of this study is that different methods were used to collect dietary data in each country. Different methods of collecting dietary data are prone to different types of bias based on in part to the respondents’ ability to recall the previous day’s intake in 24-hour recalls and to the respondents’ training to ensure an adequate level of details in describing intake in food records [13]. Food records require the individual to record food intake at the time the foods are eaten to minimize reliance on memory. However, they may alter eating behavior by reducing quantities or altering food choices [13].

Another limitation is that both recalls and food records are known to underestimate intakes. In order to decrease these errors, the AMPM uses multiple memory cues and standardized wording to help the respondent remember the previous day’s intake and includes specific probes to elicit details unique for each food or beverage. A validation study of this method shows that AMPM reduces bias in the collection of energy intake [14]. In Brazil, respondents received face-to-face training and manuals to guide them in the details required to accurately describe the foods and amounts consumed. All records were reviewed by trained interviewers to clarify entries and probe for usually forgotten foods.

This study is the first to use national dietary surveys to compare Brazilian and American diets. Although no comparisons were done before, the 2005 Dietary Guidelines for Americans and the MyPyramid were used as a template to build Brazilian national dietary guidelines [37]. These analyses allowed identification of differences in food intake that must be considered in the development of recommendations on limiting or promoting the intake of specific food categories. It also indicated that although the diet is still characterized by traditional foods, Brazilians are incorporating a more Western diet, suggesting that both the amount of food and the quality of the diet may have an important role in the increasing rates of obesity. Brazilian data indicate that there is a mosaic of traditional and industrialized items mainly among young adults. In both countries, young adults had higher intake of high-calorie and nutrient-poor foods than older adults and should be targeted for specific dietary recommendations.

## Conclusion

The Brazilian diet is different from the American diet, indicating that it is still possible to reverse the current trends of incorporating Western dietary habits by encouraging the consumption of traditional staple foods and discouraging the consumption of high-calorie and nutrient-poor foods , such as cookies, savory snacks, deli and cured meats, and soft drinks. The Western dietary pattern has been associated to obesity, diabetes and hypertension [29,30]; however, whether preventing the incorporation of Western dietary habits will decrease obesity levels warrants further examination. Longitudinal studies and/or time trend studies would allow better interpretation of the influence of the diet on the population’s weight.

## Competing interests

The authors declare that they have no competing interests.

## Authors’ contributions

INB was responsible for the study design, data analysis and interpretation and manuscript preparation. DNC, CLM, RSS, and JKA were responsible for grouping foods in food categories. DGR, MKH, and AMS were responsible for grouping foods in food categories, data interpretation and manuscript preparation. JG conducted data analysis. AJM and RS were responsible for the study design and manuscript preparation. All authors have read and approved the final manuscript.
